# Measuring proteins with greater speed and resolution while reducing sample size

**DOI:** 10.1038/s41598-017-09051-1

**Published:** 2017-08-30

**Authors:** Vincent H. Hsieh, Philip J. Wyatt

**Affiliations:** Wyatt Technology Corporation, 6330 Hollister Avenue, Goleta, California 93117 USA

## Abstract

A multi-angle light scattering (MALS) system, combined with chromatographic separation, directly measures the absolute molar mass, size and concentration of the eluate species. The measurement of these crucial properties in solution is essential in basic macromolecular characterization and all research and production stages of bio-therapeutic products. We developed a new MALS methodology that has overcome the long-standing, stubborn barrier to microliter-scale peak volumes and achieved the highest resolution and signal-to-noise performance of any MALS measurement. The novel design simultaneously facilitates online dynamic light scattering (DLS) measurements. As National Institute of Standards and Technology (NIST) new protein standard reference material (SRM 8671) is becoming the benchmark molecule against which many biomolecular analytical techniques are assessed and evaluated, we present its measurement results as a demonstration of the unique capability of our system to swiftly resolve and measure sharp (20~25 µL full-width-half-maximum) chromatography peaks. Precise measurements of protein mass and size can be accomplished 10 times faster than before with improved resolution. In the meantime the sample amount required for such measurements is reduced commensurately. These abilities will have far-reaching impacts at every stage of the development and production of biologics and bio-therapeutic formulations.

## Introduction

The unprecedented growth of biotechnology has resulted in the development and commercial production of a variety of therapeutic products including vaccines, monoclonal antibodies, biosimilars, recombinant proteins and other biologicals. Size-exclusion high-performance liquid chromatography^1, 2^ (SE-HPLC), which separates biomolecules and polymers based on their hydrodynamic volume, has long been a valued analytical tool for the determination of sample purity and presence of high molecular weight (HMW) and low molecular weight (LMW) species in bio-therapeutic formulations. Complemented with MALS detectors, the SE-HPLC-MALS system becomes a powerful analytical workhorse capable of absolute on-line molar mass measurements without the uncertainties associated with molecular calibration standards.

Associated with its release of Standard Reference Material 8671^3^, or NIST mAb, NIST subjected the antibody standard to a variety of analytical measurements and characterization. MALS^4–6^ was employed as one of the most important means for measuring the molar mass of the antibody following separation by SE-HPLC and the details have been presented in the 426-page document^7^ “Volume 2. Biopharmaceutical Characterization: The NISTmAb Case study.” Such measurements were confirmed by a variety of other orthogonal analytical instruments including mass spectrometry^8^ and sedimentation equilibrium analytical ultracentrifugation^9, 10^. In addition, dynamic light scattering^11^ (DLS), another light scattering technique, was used to measure the translational diffusion coefficient and calculate the hydrodynamic radius of the antibody.

Recent advances in column technology ushered in size-exclusion *ultra*-high-performance liquid chromatography^12^ (SE-UHPLC) and achieved an order-of-magnitude improvement in separation speed, efficiency and resolution over SE-HPLC. However, due to its large system volume, conventional MALS instrumentation introduces excessive peak broadening and could not benefit from the much-prized peak resolution of SE-UHPLC. In the NIST report, the SRM 8671 standard was separated by SE-UHPLC and the UV concentration detector disclosed the presence of both HMW species (~2% mass fraction) and a lower molar mass fragment (~0.1% mass fraction) in addition to the monomeric component. Other means had to be used to determine the molar mass of both the antibody and the fragment; the preferred MALS and DLS measurements could not be made satisfactorily because conventional MALS instrumentation introduced excessive peak dispersion and was incompatible with SE-UHPLC.

In this paper, we show a new MALS and online DLS^13^ design that is fully compatible with SE-UHPLC system. With the new flow cell design and associated optics, it is now possible to *directly* measure the molar masses of eluted species, including the antibody monomer and the accompanying fragment, with uncompromised peak resolution. The new detection concept is referred to as “micro-MALS”, or simply μMALS since the MALS measurements may now be made on small microliter fractions characteristic of the UHPLC separations.

## Results and Discussion

### MALS System

**Figure 1:**
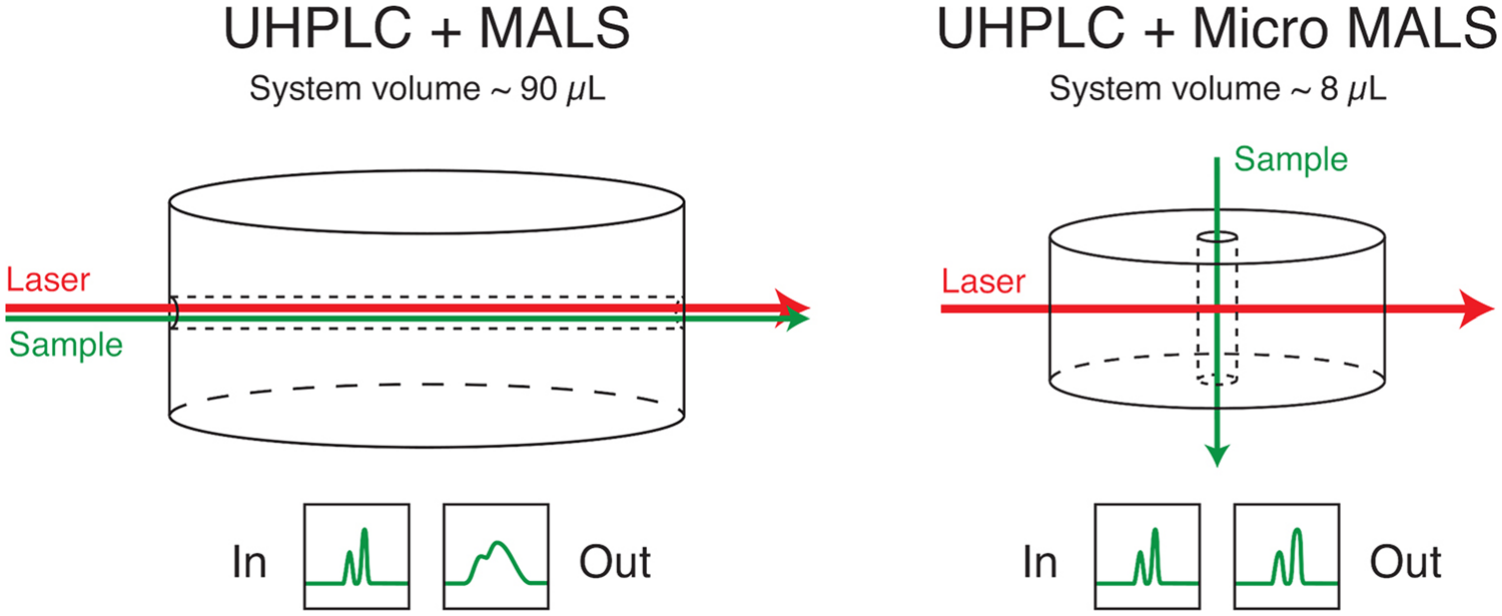
Comparison between the µMALS and MALS flow cells geometries and system volumes. The much smaller volume of the µMALS system has significantly reduced the peak dispersion and is the most important contributing factor to the µMALS compatibility with SE-UHPLC whose typical peak FWHM width is 20~25 µL. The traditional MALS system introduces too much dispersion and degrades resolution of narrowly-resolved peaks.

Flow path in the traditional MALS instruments has a typical system volume of ~100 µL, including the flow cell and inlet/outlet tubings. While such a system volume is certainly compatible with peak volumes (~250 µL, FWHM) eluted from SE-HPLC, it is too large to adequately resolve a typical SE-UHPLC elution peak (~25 µL, FWHM). With the new cell design and implementation of MALS optics, we were successful in reducing the total system volume from 90 µL to 8 µL by adopting a different fluid flow and optical geometry, as shown in Fig. 1. This is the first instance of a high-performance MALS system with a total volume less than 10 µL. Both the flow cell and tubing volumes were reduced by approximately an order of magnitude to achieve this significant improvement. As a result, the dispersion of the µMALS system has also decreased by approximately an order of magnitude compared with the traditional MALS instrumentation. Figure 1 illustrates how the reduction in peak dispersion in the µMALS system dramatically improves the peak resolution over that of the conventional MALS systems. Narrowly separated molecular species remain well resolved all through the µMALS detector. (Cf. right side of Fig. 1.) This renders µMALS system compatible with SE-UHPLC.

One of the major differences between the new µMALS system and a conventional MALS system is that the laser beam direction is perpendicular to the fluid flow. The optical system was necessarily redesigned to effectively reject the stray light scattered from the now closer interfaces. Additionally, a DLS detector is incorporated into the µMALS optical system so that the hydrodynamic radius of the analytes can be measured simultaneously. Adopting a back-scattering configuration, this improves the DLS sensitivity and extends measureable hydrodynamic size range at any given flow rate.

### Experiment Results

**Figure 2:**
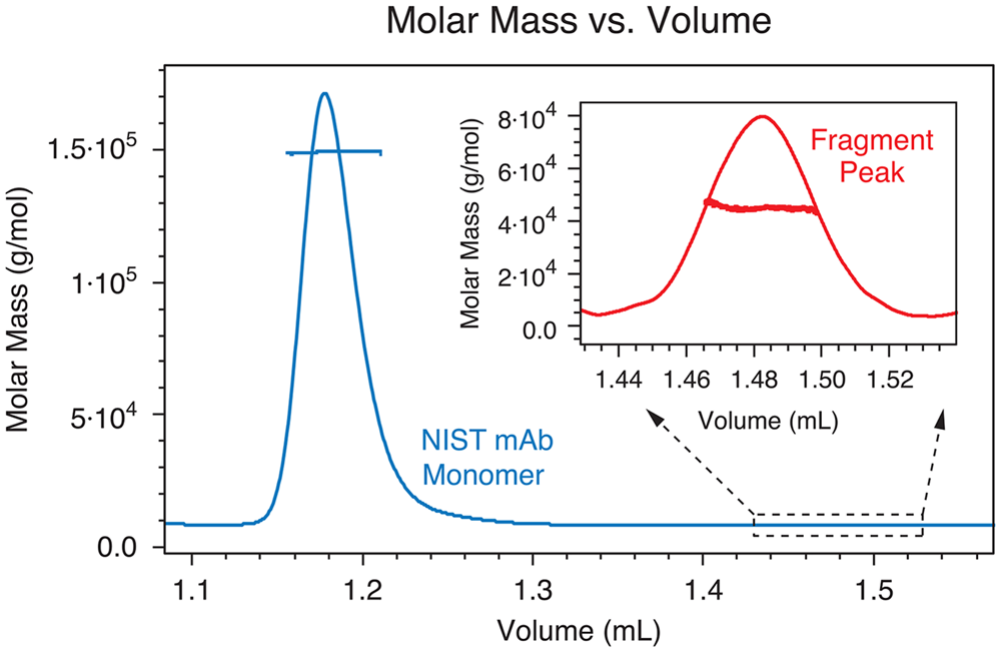
Chromatogram of the SE-UHPLC-µMALS measurement of NIST mAb monoclonal antibody. Molar mass results are overlaid on light scattering signals at 90° angle of detection. The inset further shows a separated and measured fragment peak.

Triplicates of µMALS measurements are made at 45°, 90°, and 135° of NIST mAb after fractionation by SE-UHPLC. Following the μMALS detector, the mass concentration of the monoclonal antibody and the small fraction were determined by a differential refractometer using a *dn/dc* value of 0.187 mL/g. This value was obtained by comparing the peak mass recovery by both UV (with an extinction coefficient^1^ of 1.42 mL ∙ mg^−1^ ∙ cm^−1^) and the differential refractometer. The 90° scattering signal shown in Fig. 2 is overlaid with the measured molar mass of the monomeric component of the NIST monoclonal antibody. The figure’s inset further shows an extremely small additional mass fraction not discernible in the corresponding SE-HPLC-MALS measurements reported in the NIST study.

Consequently, the μMALS system was able to easily resolve and directly measure molar masses of the monomeric and fragment components of the monoclonal antibody: the monomer has a solution molar mass of *149.1* kDa ± 0.3% and the fragment’s solution molar mass is *44.9* kDa ± 8.5%. It should be noted here that the calculated molar mass of the fragment peak is based on the assumption that its *dn/dc* value is the same as the monomeric component.

The measured monomer molar mass is in excellent agreement with the value derived from the far more labor-intensive mass spectrometry^5^. Interestingly, the mass fraction of the fragment was very stable and unaffected by the flow rate of the SE-UHPLC system. This measurement was remarkable on two additional accounts: 1) The total elution volume was only about 1.2 mL or roughly 1/7 that of the traditional SE-HPLC measurement; 2) the molar masses of the two distinct molecular species spanned a concentration range of three orders of magnitude from approximately 1.0 mg/mL to 1.0 µg/mL, yet were still easily detected and measured. This highlights the exceptional dynamic range of µMALS detection in addition to its resolution.

**Figure 3:**
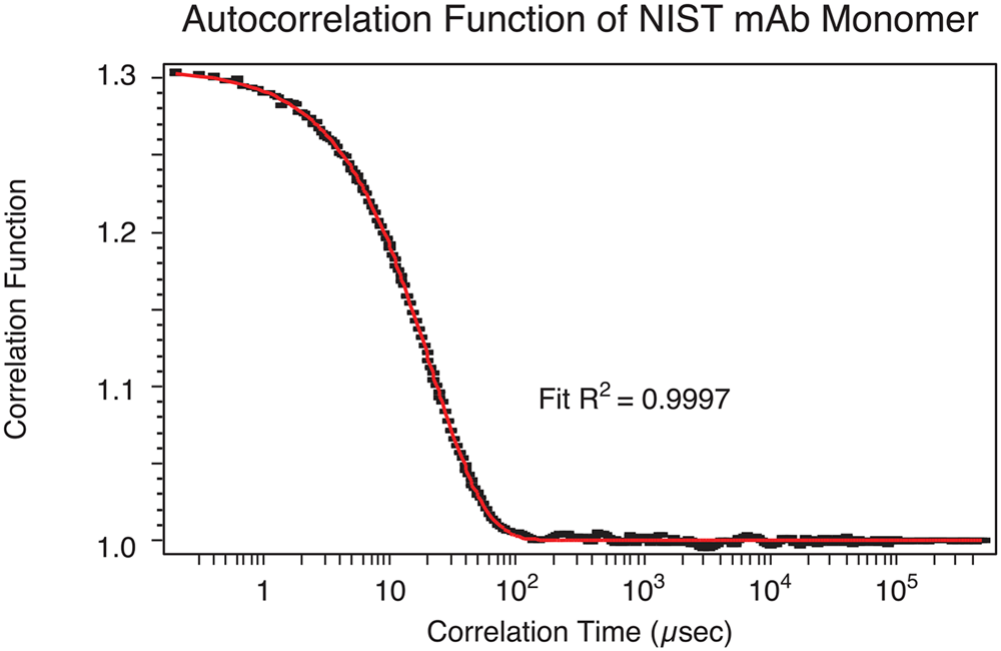
The autocorrelation function and curve fit of the NIST mAb monomer peak. The *online* DLS measurement is taken at a scattering angle of 130°.

The on-line DLS measurement, *simultaneously* made in the same μMALS cell, yielded the hydrodynamic radius value, 5.5 nm ± 1.5%, for the monomer peak. It agrees exceedingly well with the monomer radius (5.50 nm) published by NIST based on the ultracentrifugation sedimentation coefficient and solution molar mass^5^. Shown below in Fig. 3 is the measured autocorrelation function and fit of the eluted monomer peak. The correlation decay time measures the translational diffusion coefficient. The hydrodynamic radius is then calculated through the Stokes-Einstein relationship. The hydrodynamic radius of the fragment could not be measured because of its very low concentration, but may be estimated to be 3.7 nm by comparing the radii of two equivalent density spheres, *i. e*.

$${{{\rm{R}}}_{{\rm{h}}}}^{3}/44.9={5.5}^{3}/149.1,{\rm{thus}}\,{R}_{{\rm{h}}} \sim 3.7\,{\rm{nm}}.$$

**Figure 4:**
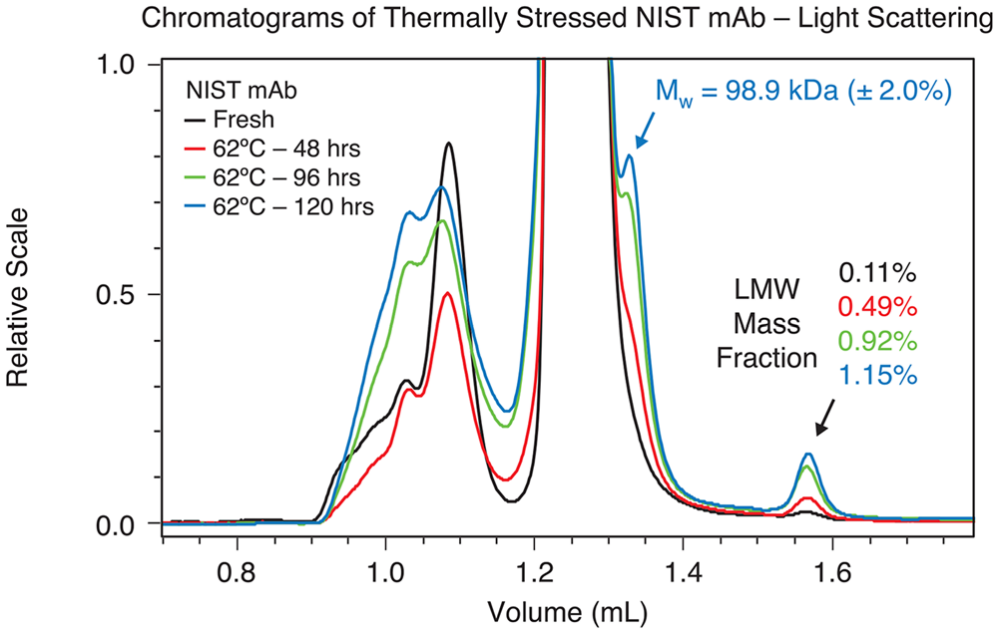
Comparison between chromatograms of un-stressed and thermally stressed NIST mAb samples. Incubation of sample at elevated temperature increases the mass fraction of both the HMW and LMW species. The µMALS system determined the molar mass and mass fraction of each species of interest.

**Figure 5:**
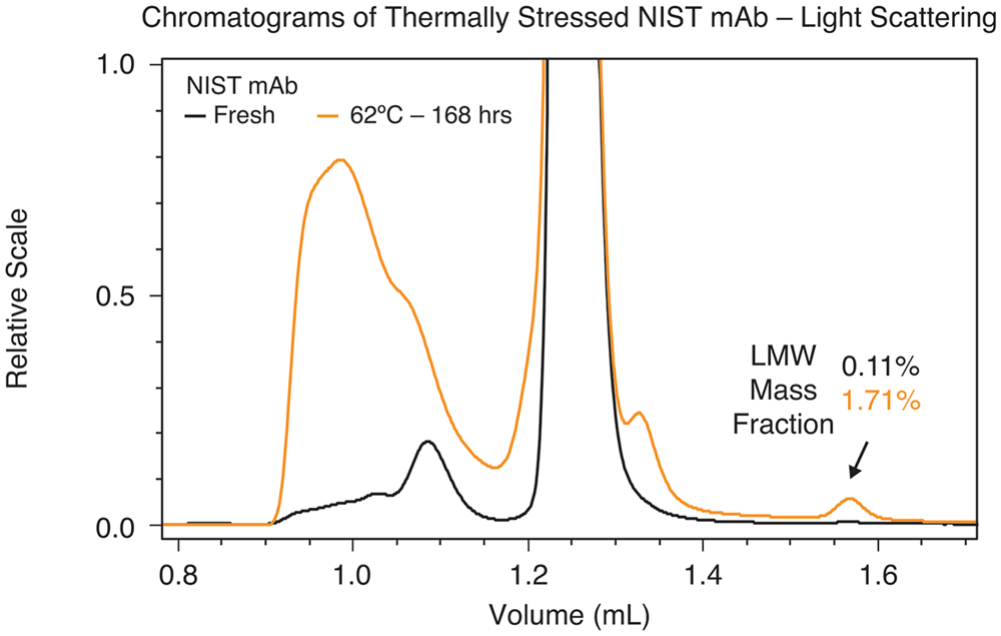
After 168 hours of incubation at 62 °C, the mass fraction of HMW species have a more dramatic increase, from 2.0% to 10.4%. The µMALS system determines the weight-average molar mass of the HMW to have increased from 387.7 kDa ± 2.2% to 476.3 kDa ± 0.90%.

To further demonstrate the utility of µMALS and applications in characterizing sample purity and the presence of HMW and LMW species, the NIST mAb sample was subjected to accelerated thermal degradation experiment by incubation at 62 °C up to 7 days. We chose to incubate the sample at a temperature slightly above its T_onset_ (≈ 60 °C) in its formulation buffer^7^. Every day, triplicate SE-UHPLC-µMALS measurements are carried out along with measurements of unstressed sample, kept at 4 °C, as control. Figures 4 and  5 show these interesting results:

1. The mass fraction of the LMW species which elutes around 1.5 mL increases with accumulating thermal stress, from ~0.1% in a fresh sample to more than 1.1% after 120 hours’ incubation at 62 °C.
2. A second LMW species, which elutes around 1.35 mL, becomes increasingly conspicuous following the accumulation of thermal stress. Assuming the same *dn/dc* value as the monomer, its molar mass is measured as 98.9 kDa.
3. The mass fraction of the HMW species began to increase more dramatically after more than 120 hours of heat stress. After 168 hours of incubation, the mass fraction of the HMW species increased almost five-fold to 10.4%. Additionally, the weight-average molar mass of the HMW portion increased from 387.7 kDa (±2.2%) for the unstressed sample to 476.3 kDa (±0.9%).

The successful measurement of the obscure antibody ‘fragment’ peak is a testament to the unprecedented sensitivity of the instrumentation. On top of a significant improvement in speed and a corresponding decrease in sample consumption, the µMALS embodiment is shown to deliver enhanced resolution and sensitivity for online molar mass measurement. Furthermore, with the incorporation of dynamic light scattering (DLS), we have shown that the corresponding molecular size may be measured simultaneously. Thus, research scientists will be able to detect and assess with far greater precision such critical molecular properties as monomeric purity, aggregate formation, fragment identification, etc.

## Conclusion

Based on first principles, MALS has long been prized as an analytical technique that is able to measure directly the absolute molar mass and size of macromolecules in solution. Its unique capability is brought into even greater utility when the sample of interest is subjected to fractionation by size exclusion chromatography before passing into MALS detector for inline molar mass measurement. With recent advancements in the field of UHPLC, the sample components are separated with much greater resolution and speed. This leaves conventional MALS instrumentation wanting especially in the aspect of elution peak dispersion.

Our work here presents a break-through development that immediately solves this clear and present analytical need for the first time. Unseen molecular species can now be revealed and differences between therapeutic agents from different batches or sources can be disclosed more rapidly and accurately. The synergistic union of µMALS and SE-UHPLC will have a profound impact at every stage of the development and production of biologicals and bio-therapeutic formulations for years to come.

## Methods

### Experimental Conditions and Setup

The sample used was NIST standard reference material 8671 (Lot No. 14HB-D-001), a recombinant humanized IgG1κ monoclonal antibody.

SE-UHPLC was performed with an Acquity UPLC pump and autosampler (Waters Corporation) and Acquity UPLC® Protein BEH SEC column (200 Å, 1.7 µm, 4.6 mm × 150 mm, Waters Corporation). The mobile phase was 100 mM sodium phosphate pH 6.8, supplemented with 250 mM NaCl. Samples were injected onto a pre-conditioned column at a flow rate of 0.300 mL/min. Triplicate injections of 1 µL, 2 µL and 6 µL of samples were made to confirm reproducibility. The effluent of the SEC column flowed through an inline UV/Vis detector (Waters Corporation), the new MALS/DLS detector and an Optilab UT-rEX dRI detector (Wyatt Technology Corporation). For the thermal stress study, triplicate injections of 1 µL of the sample were made. Unstressed sample kept at 4 °C is measured as a control each time measurements are made of the heat-stressed sample.

Data collection and analysis was performed with ASTRA software, version 7.1.1 (Wyatt Technology Corporation). ASTRA software utilizes a patented method^14^ for the interdetector delay and band broadening correction. First-order fit Zimm formalism is used for all molar mass calculation.

### Data Availability

Data files are available upon request from the corresponding author.
